# The Carotid Sinus as a Viscometer

**DOI:** 10.3390/diagnostics10110924

**Published:** 2020-11-10

**Authors:** Andrew Iskander, Rotem Naftalovich, Niema M. Pahlevan

**Affiliations:** 1 Department of Anesthesiology, Westchester Medical Center, 100 Woods Rd, Valhalla, NY 10595, USA; 2 Department of Anesthesiology, Rutgers, New Jersey Medical School, 185 S. Orange Avenue, MSB E-538, Newark, NJ 07103, USA; naftalro@njms.rutgers.edu; 3 Division of Cardiovascular Medicine, Keck School of Medicine, University of Southern California, Los Angeles, CA 90089, USA; pahlevan@usc.edu; 4 Department of Aerospace & Mechanical Engineering, University of Southern California, Rm 470, Michelson Bldg., 1002 Childs Way, Los Angeles, CA 90089, USA

Our group thought the study by Lee and Kim entitled “Hemodynamic Changes in the Carotid Artery after Infusion of Normal Saline Using Computational Fluid Dynamics” was a very elegant method to discern the changes in blood rheology within the carotid sinus after administration of crystalloid [1]. The carotid sinus is understood to be one of the regions in the body responsible for reception of information utilized by the cardiovascular system to maintain perfusion homeostasis. Rheological homeostasis is itself a complex, real-time, dynamic system that entails adjustment of various parameters responsible for maintenance of end-organ perfusion [2]. This bulb-shaped region in the proximal internal carotid just distal to the bifurcation is one of the primary sites where changes in rheology exerted on the endothelium alter the firing of neural signals along the nerve of Hering to the brainstem. This data is then used to adjust blood delivery over the course of the cardiac cycle and degree of vasomotor tone throughout the vascular tree, resulting in beat-to-beat adjustment of the end-organ blood pressure.

The relationship of the carotid sinus to blood rheology is analogous to that of the ear to sound. One can consider the external ear as a resonator tube [3]. As sound passes through this tube, gain is added thereby amplifying and enabling the composite frequencies of sound to be more discernable by the middle and inner ear and ultimately processed by the central nervous system. Sound, therefore, can be transduced to the central nervous system for the auditory environment to be perceived. Similarly, we maintain that the anatomy and location of the carotid sinus serve as an optimal location to discern the rheological character of blood—particularly changes in viscosity. The dilation at the origin of the internal carotid—comprising the sinus region—allows space for swirling and the development of secondary flows in what is referred to as the region of recirculation [4]. This region of shear oscillation, described by Ku, allows for an amplifying of the rheological properties of blood flow that depend on blood viscosity, itself an important factor in the thixotropic effects of blood flow in large conducting vessels and capillary beds. In effect, “gain” is added to the viscometric character of blood. 

In our computer fluid dynamics depiction of wall shear stress of a carotid bifurcation [5] (see Figure 1), the changes in sheer stress over the cardiac cycle are largest at precisely the region innervated by the carotid sinus nerve. The magnitude of the sheer stress changes seen in that region are themselves a direct correlate of the blood viscosity. Lee and Kim describe a decrease in wall shear stress and an increase in shear rate with decreased viscosity after saline infusion. Remarkably, the PIEZO receptor population recently described [6] in the vessel wall using a murine model of carotid sinus knock-out makes this region particularly well-suited to detecting even small changes in the shearing behavior of blood as it recirculates in the region of low wall shear stress. We reason that the location of the carotid sinus, in the context of its physical anatomy and receptor population, supports the hypothesis that the sinus region acts as a transducer of blood viscosity.

## Figures and Tables

**Figure 1:**
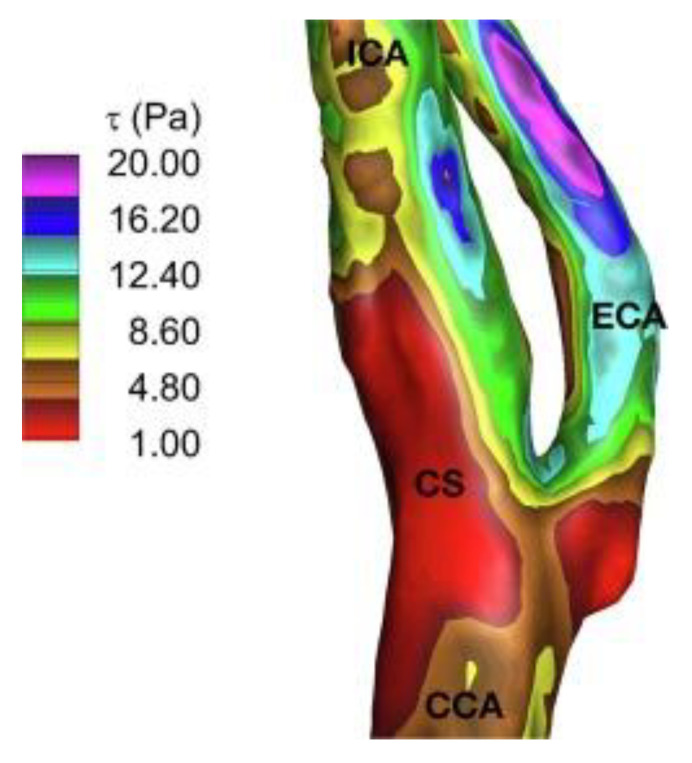
Figure depicting the location of the carotid sinus (CS) in the region of lowest wall shear stress. (ECA = external carotid artery, ICA = internal carotid artery, CC = common carotid artery.
